# Post-Translational Modifications of Cytochrome *c* in Cell Life and Disease

**DOI:** 10.3390/ijms21228483

**Published:** 2020-11-11

**Authors:** Alejandra Guerra-Castellano, Inmaculada Márquez, Gonzalo Pérez-Mejías, Antonio Díaz-Quintana, Miguel A. De la Rosa, Irene Díaz-Moreno

**Affiliations:** Instituto de Investigaciones Químicas (IIQ), Centro de Investigaciones Científicas Isla de la Cartuja (cicCartuja), Universidad de Sevilla, Consejo Superior de Investigaciones Científicas (CSIC), Avda. Américo Vespucio 49, 41092 Sevilla, Spain; alejandra.guerra@iiq.csic.es (A.G.-C.); imarquez1@us.es (I.M.); gonzalo.perez@iiq.csic.es (G.P.-M.); qzaida@us.es (A.D.-Q.); marosa@us.es (M.A.D.l.R.)

**Keywords:** cytochrome *c*, mitochondrial diseases, post-translational modifications

## Abstract

Mitochondria are the powerhouses of the cell, whilst their malfunction is related to several human pathologies, including neurodegenerative diseases, cardiovascular diseases, and various types of cancer. In mitochondrial metabolism, cytochrome *c* is a small soluble heme protein that acts as an essential redox carrier in the respiratory electron transport chain. However, cytochrome *c* is likewise an essential protein in the cytoplasm acting as an activator of programmed cell death. Such a dual role of cytochrome *c* in cell life and death is indeed fine-regulated by a wide variety of protein post-translational modifications. In this work, we show how these modifications can alter cytochrome *c* structure and functionality, thus emerging as a control mechanism of cell metabolism but also as a key element in development and prevention of pathologies.

## 1. Introduction

Mitochondria are the powerhouses of the cell. Their dysfunction favors the development of neurodegenerative diseases, cardiovascular pathologies, and several types of cancer [1]. A key protein in mitochondrial metabolism and control of redox signaling is cytochrome *c* (C*c*) [2]. C*c* is a small soluble protein (*ca*. 12 kDa, 104 amino acids) with four α-helices and a heme group, which is covalently bound by two cysteine residues (Cys14 and Cys17). The heme group is wrapped in a hydrophobic crevice, being only slightly exposed to the solvent. This conformation allows C*c* to efficiently exchange electrons with its redox partners. Several residues of C*c* undergo post-translational modifications, which provide a fine-tuned regulation at the functional level [3]. In the absence of stimuli, C*c* is located in the mitochondrial intermembrane space, participating in redox metabolism [4]. However, several stimuli—such as apoptotic or DNA damage signals—promote C*c* release from mitochondria. The first extramitochondrial target reported for C*c* was Apoptosis protease-activating factor-1 (Apaf-1) [5]. The interaction of C*c* with Apaf-1 in the cytosol is an early event of the mitochondrial apoptotic pathway [3]. Actually, the mitochondrial and extramitochondrial network of C*c* encompasses a variety of proteins that are located in different organelles and cytosol [3,6,7]. These interactions can modulate the cell fate decision between cell life and death [7], which in turn relate to health and disease. The objective of this review is to carry out an in-depth analysis of how C*c* modifications affect its functions and their impact on the development of certain diseases and pathologies.

## 2. The Pleiotropic Role of Cytochrome *c* in Cell Homeostasis and Diseases

**Figure 1:**
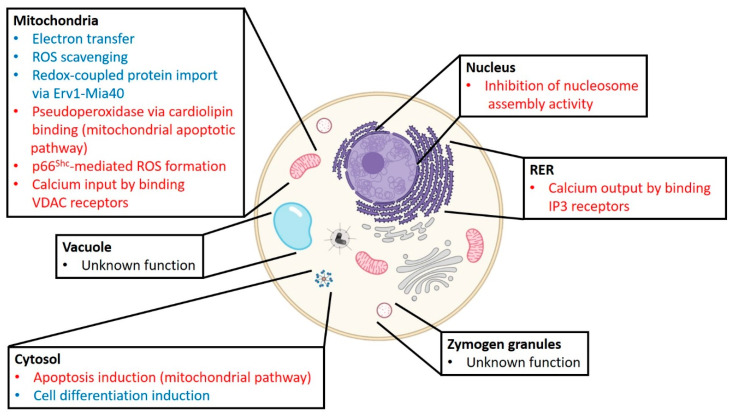
Cell localization and functions of cytochrome *c*. C*c* is located in the mitochondrial intermembrane space, vacuole and zymogen granules under homeostasis. However, during DNA damage and apoptosis stimuli, mitochondrial C*c* travels into the nucleus, the rough endoplasmic reticulum (RER) and the cytoplasm, respectively. The most relevant functions performed by C*c* in each location are explained in the boxes. Color key: blue, physiological functions; red, functions performed under stress; and black, unknown functions. Created with BioRender.com (https://biorender.com/).

C*c* is a moonlighting protein whose localization and functions depend on cellular conditions (Figure 1) [3,6,7]. Under homeostasis, it is a key component of cellular energy metabolism, acting as an electron carrier between the cytochrome *bc*_1_ complex (complex III, CIII) and cytochrome *c* oxidase (complex III, CIV) in the mitochondrial electron transport chain (ETC) [4]. In this context, two binding sites have been described in cytochrome *c*_1_ (C*c*_1_; from CIII) and CIV for C*c*: a *proximal* site, optimal for electron transfer, and a *distal* site, which is not productive in terms of electronic transfer. It has been postulated that the interaction at the *distal* site could have physiological relevance in the dynamics and organization of electronic flow. It also seems to increase the local concentration of C*c* available near the *proximal* site, allowing a rapid turnover of C*c* molecules [8,9]. Recently, the molecular basis of electron transfer from C*c*_1_ in CIII to C*c* has been described [10,11]. According to the data, the coupling of redox potential shifts the conformational cycle of the Rieske subunit and the binding of C*c* to C*c*_1_ causes electrons to flow in a single direction [11]. C*c* has other redox functions, acting as a reactive oxygen species (ROS) scavenger and participating in the import of redox-coupled cysteine-rich proteins via Erv1-Mia40 [12,13,14]. The heme protein is also localized in vacuoles and zymogen granules during cell homeostasis, although its role there is unknown [15].

Under nitro-oxidative stress conditions, C*c* contributes to ROS production via the p66 redox cycle and acts as an inducer of programmed cell death (PCD) (Figure 1) [16]. Alterations in PCD signaling are relevant in many diseases [17,18]. Recent data indicates that the apoptotic network involving C*c* is complex, and several target proteins are functionally equivalent in humans, plants, and *Drosophila melanogaster* [19,20,21,22,23,24,25]. At the onset of apoptosis, a C*c* population—tightly bound to the inner mitochondrial membrane—catalyzes peroxidation of phospholipids, particularly cardiolipin (CL) (Figure 1). The nature of the C*c*/CL interaction is subject of intense research [26]. Some authors proposed that CL-adducted C*c* undergoes a profound tertiary conformational rearrangement that opens an entry channel for H_2_O_2_ molecules, enhancing the peroxidase activity of C*c* [5,27]. Extensive data in the literature hints that the interaction with the CL affects the dynamics and conformation of a set of loops that determine the heme environment and iron coordination of C*c*. In particular, the bond between the Sδ atom of Met80 and iron can be disrupted, leading to high spin penta-coordinated species resembling that described for myoglobin [28]. It has been proposed that C*c*/CL conjugates are sufficient for the formation of mitochondrial pores, which allow the release of the hemeprotein into the cytosol during apoptosis and redistribution of CL from the inner to the outer mitochondrial membrane [29,30]. In the early stages of apoptosis, C*c* interacts with the inositol 1,4,5-trisphosphate (IP3) receptor in the rough endoplasmic reticulum (RER) and with the voltage-dependent anion channel (VDAC) in the outer mitochondrial membrane. These interactions, in turn, promote calcium output and input, respectively (Figure 1) [31,32,33]. This traffic of calcium ions is restricted in space since both the IP3 receptors and the VDAC are located in mitochondria-associated endoplasmic reticulum membranes (MAMs) [34,35]. MAMs are microdomains involving both RER and mitochondrial proteins. These interconnection zones have fundamental roles in the transmission of calcium ions but are closely related to cellular lipid and energy metabolism [36,37]. The interaction of C*c* with the IP3 receptor prevents its calcium-induced inhibition, triggering the release of calcium [38]. Calcium binds to VDAC dimers, resulting in oligomerization [39]. This event increases the conductance of the channel and allows the release of C*c* through it [40,41,42]. It is worth mentioning that the release of C*c* into the cytosol may not always lead to cell death, and the heme protein can sometimes act as a cell differentiation inductor (Figure 1) [20,43]. C*c* also reaches the cell nucleus upon DNA damage where it sequesters histone chaperones and inhibits chromatin remodeling (Figure 1) [23,24]. The pleiotropy of the effects of extramitochondrial C*c* can be modulated by the cytosolic diflavin reductase NDOR1, which has NADPH-dependent C*c* reductase activity [44].

## 3. Post-Translational Modifications of Cytochrome *c*: Regulation, Functionality, and Structural Changes

Post-translational modifications (PTMs) in proteins are regulatory mechanisms which control an ample set of cell metabolic processes and provide a tool to increase the functional diversity of proteins [45,46,47]. Indeed, PTMs play an integral role in regulating C*c* functions [48,49]. The heme protein can undergo different PTMs (see below) that affect its physicochemical properties, interactions with physiological partners and, consequently, its functions. However, these effects depend on which residues are post-translationally modified.

### 3.1. Phosphorylation

The functionality of C*c* is controlled in vivo by phosphorylation of the following residues: Thr28, Ser47, Tyr48, Thr58, and Tyr97 [50,51,52,53,54,55]. Since Thr58 is replaced by isoleucine in humans, this position will not be a subject of this review. The low yield of phosphorylated C*c* during purification procedures makes functional and structural analysis challenging. Furthermore, the specific kinases responsible for phosphorylating the protein remain unknown—although some authors suggest that it could be the AMP kinase [53]. Therefore, it is common to mimic targeted phosphorylation with site-directed mutations that encode canonical or non-canonical (AMBER-type stop codons) substitutions. The evolved tRNA synthetase technique allows the site-specific incorporation of a non-canonical amino acid into the protein sequence [56]. The method is based on the use of an orthogonal aminoacyl-tRNA synthetases/tRNA pair. The aminoacyl-tRNA synthetase identifies the non-canonical amino acid and charges it in the tRNA. This is a special tRNA that recognizes AMBER-type stop codons, allowing the introduction of the residue into the desired position. The evolved tRNA synthetase technique can be used for the incorporation of non-canonical amino acids into proteins being produced in both prokaryotic and eukaryotic cells [57,58,59].

The use of non-canonical amino acids aims at a better emulating the physical properties of the target residues—e.g., the volume and/or charge—as compared to canonical substitutions (Table S1) [60,61,62,63,64,65,66,67,68,69,70,71,72,73,74,75,76,77]. A good example is the non-natural residue p-carboxymethyl-L-phenylalanine (pCMF), which has been widely used to mimic tyrosine phosphorylation [77]. Non-canonical amino acids are indeed resistant to hydrolysis, making them ideal candidates for use in pharmacological therapy (see below).

The structural and functional effects of phosphorylations at positions 28 and 47 have been studied using samples of C*c* both, phosphorylated in vivo and via the use of phosphomimetic species. The isolated C*c* in vivo phosphorylated at positions 28 and 47 donates electrons to CIV less efficiently than the wild-type species [53,55]. Phosphomimetic species at Thr28 show enhanced peroxidase activity at a low C*c*/CL ratio [52,77]. This function correlates with the ability of C*c* to be translocated from the mitochondria into the cytoplasm, where it triggers apoptosis. In addition, mutations at Ser47 impairs C*c*–mediated caspase cascade activation, which is an essential step proceeding programmed cell death (Table 1) [55,78].

In relation to tyrosine phosphorylation, the presence of negative charges at position 48 affects the redox properties of C*c* and lowers the p*K*_a_ value of the alkaline transition towards physiological pH values [77,79,80]. In turn, in vivo phosphorylated C*c* and its phosphomimetics at position 48 diminish oxygen consumption [51,79,81]. In fact, during supercomplex formation the Y48pCMF mutant shows decreased electron transfer to CIV. This finding was attributed to the lower binding affinity of the aforementioned mutant for the distal sites of C*c*_1_ (in CIII) and CIV. These changes in binding equilibria alter the preferential diffusion pathway that channels C*c* molecules through the CIII/CIV supercomplex [81]. On the other hand, the phosphomimetic species display a higher ROS scavenger activity and a more efficient peroxidase activity when binding to CL [79,80,81]. Phosphorylation also alters the apoptotic function of C*c* by inhibiting its ability to activate the caspase cascade [79,80,81]. The mutant Y97pCMF C*c* likewise displays decreased electron transfer to CIV in the context of supercomplex formation, despite no substantial changes in C*c* peroxidase activity. However, Y97pCMF C*c* acts as an inefficient caspase activator [82].

### 3.2. Nitration and Nitrosylation

The effects of tyrosine nitration and nitrosylation in C*c* have been studied extensively due to their roles in cell metabolism under stress [85,86,87,119,120,121,122]. Nitric oxide (NO) is a signaling molecule with pleiotropic effects. A significant number of these effects are exerted through PTMs mediated by nitric oxide-derived reactive nitrogen species (RNS) under physiological and stress conditions [123]. Nitration and nitrosylation consist of non-enzymatic covalent modifications that introduce a nitro group (-NO_2_) at the phenolic ring of tyrosine or a nitrosyl group (-NO) at the thiol group of cysteine residues, respectively. C*c* has no free cysteines—Cys14 and Cys17 are covalently linked to the heme group—but the Met80 axial ligand and/or heme Fe atom are susceptible to nitrosylation [124]. -NO_2_ and -NO primarily originate from RNS, such as peroxynitrite [119,125]. However, recent studies have shown that these modifications can be produced by diffusion of the NO radical into the mitochondria [126].

Nitration of C*c* tyrosine residues 46 and 48 has not been observed in vivo. In fact, Díaz-Moreno and co-workers showed that the nitration of these residues enhances proteolytic degradation of C*c* by cell extracts [83]. In vitro assays demonstrate that nitration of any tyrosine from C*c* increases its peroxidase activity and impairs membrane potential formation [84]. The impact on the redox properties of C*c* increases with the number of nitrated tyrosines [126,127]. This explains why electron transfer increases when C*c* is doubly nitrated [128,129] (Table 1). Notably, nitration at Tyr46, Tyr48, and Tyr74 decreases the p*K*_a_ value of C*c* alkaline transition [83,86].

On the other hand, nitrosylation of C*c* induces changes in protein conformation and heme configuration/coordination [89]. Such effects on redox C*c* properties inhibit its peroxidase activity and enhance its ability to activate the caspase cascade. For this reason, it has been proposed that C*c* nitrosylation is a proapoptotic modification [88,89,124].

### 3.3. Acetylation

C*c* acetylation has been studied for decades after Minakami et al. first explored the properties of acetylated C*c* [91]. Acetylation of lysine residues is an enzymatic process carried out by lysine acetyltransferases [130]. However, it has been observed that in some cases the acetylation process takes place via a non-enzymatic mechanism in the mitochondrial matrix, where the transfer of an acetyl group is promoted by high concentrations of acetyl-CoA and an alkaline pH [92].

In general, the acetylation of lysine residues results in a decrease of C*c*-mediated electron transfer in the respiratory chain [90], as the rate of C*c* reduction/oxidation is greatly reduced [91]. The effect on oxidation-reduction efficiency is explained by the loss of the positively charged lysine residues 72, 73 and 79 which would induce changes in the heme environment (Table 1) [93]. Conservation of the environment is crucial in preserving C*c* redox stability [91]. Likewise, changes in the p*I* value also influence the interaction of C*c* with CIII and CIV. Lower p*I* values prevent these interactions, which in turn impact on the electron transport chain [94]. This downregulation of mitochondrial electron flow causes the “Warburg effect” (cell respiration inhibition) [96]. The effect of net charge decrease is less pronounced on the native structure when the acetylated groups are located on the protein surface [92]. However, it was found that acetylation of more than six lysine residues resulted in a decrease in the overall positive charge of the protein, which plays a key role in the interaction between C*c* and CIV, leading to a complete loss of the ability of C*c* to transfer electrons to CIV [92]. Moreover, Korshunov et al. showed that in vitro acetylation of horse heart C*c* prevents ROS scavenger activity [94]. Only acetylation of Lys8 and Lys53 in mammalian C*c* has been described in vivo [96,131]. Although C*c* acetylation has been studied for decades, scope of this PTM and its cellular impact remains unknown, leaving the door open for further investigations [48].

### 3.4. Glycosylation and Glycations

Recent studies have elucidated the main features of chemical C*c* glycosylation, which comprises addition of a carbohydrate moiety to a protein molecule. Méndez et al. have studied the effect of equine heart C*c* glycosylation via an activated lactose, finding improved thermodynamic and colloidal stability of the glycosylated C*c* [97] which was resistant to protein denaturation or unfolding [132]. Glycosylation of C*c* also protects the protein surface due to the shielding effect of the glycan, which decreases the proteolytic degradation process carried out by proteases. It has also been demonstrated that that glycosylation of lysine residues leads to some minor perturbations in protein tertiary structure, whose integrity is crucial for apoptosis induction [98].

Whereas C*c* glycosylation is an enzyme-directed mechanism, glycation is a non-enzymatic chemical process [133]. The in vitro glycation of Arg91 in horse heart C*c* induces conformational changes in the protein structure due to an increase in the α-helical content which alters the secondary structure and, therefore, protein folding [99]. C*c* glycation leads to an aggregation process through monomer addition, driven by the exposition of new hydrophobic segments to the solvent [100].

Other C*c* glycation target residues include Lys72 and Lys87/88, which are located in a cationic patch involved in the association of C*c* with CL-containing membranes [101]. The glycation of these residues lowers the positive charge of the protein, hindering the interaction between C*c* and the CL membrane. The effect of C*c* glycation on the electron transfer chain highlights the importance of lysine residues on the ability of heme protein to interact with its partners [12,134,135,136].

In fact, a recent study showed that glycation of bovine heart C*c* by glyoxal caused conformational alteration in protein structure [102]. This alteration resulted in perturbation of tertiary structural interactions induced by the formation of a penta-coordinated structure that may also promote the reduction of heme, altering the redox state of C*c*. Godoy et al. have shown that it also occurs in a C*c* M80A mutant, in which the methionine ligand is replaced with an alanine residue, disrupting the Met80-Fe interaction [137]. Moreover, C*c* glycation activates peroxidase activity, which is a crucial step in the release of C*c* from mitochondria [102].

### 3.5. Deamidations

Mammalian C*c* deamidation was discovered by Flatmark in 1964 as a result of C*c* fractions with different electrophoresis mobility [138]. C*c* exposed to different pH and temperature values leads to the deamidation at glutamine and/or asparagine residues in consecutive steps [103,139]. These residues are involved in the maintenance of C*c* native structure. In particular, residues Asn31 and Gln42, Asn52 and Asn70 have a structural and/or functional role [104], thus these modifications affect the biological activity of C*c* (Table 1).

C*c* deamidation is a nonenzymatic process [140]. However, as mammalian tissues are rich in hydrolyze-amide enzymes, Flatmark’s work suggested that the in vivo deamidation of C*c* takes place by an enzymatic mechanism [141].

### 3.6. Sulfoxidation

Sulfoxidation is a PTM involving oxidation of a sulfur group on a methionine residue. Generally, this is Met80 in C*c* [108]. The key role of Met80 is to coordinate the heme center together with His18 and covalently-bound Cys14 and Cys17, maintaining a packed and hydrophobic environment for the heme iron that ensures a suitable redox potential for C*c* biological functions [109]. Oxidation of Met80 to methionine sulfoxide opens the heme coordination pocket [137], altering the protein configuration. Freeing the heme coordination could facilitate the interaction between the iron atom and lysine residues, changing the p*K*_a_ value of the alkaline transition. This change in protein configuration enables the iron atom to interact with ligands such as NO, CO, O_2_, and H_2_O_2_ [30,125]. This interaction likely disrupts the electron transport chain and restrains energy transduction in mitochondria [110].

Sulfoxidation of Met80 also enhances the peroxidase activity of C*c* [107], causing the simultaneous reduction of H_2_O_2_ and oxidization of associated CL. This diminishes electron transport as it increases heme center accessibility [105]. In fact, Rouco et al. have recently measured the binding affinity between sulfoxidized C*c* and CL, finding an increase of ca. 4 times in affinity in comparison to WT C*c* [107]. This finding would explain the enhancement of peroxidase activity shown by the sulfoxidized species. Moreover, Yin et al. have shown that Met80 sulfoxidation followed by lysine carbonylation results in an even greater C*c* peroxidase activity when compared to the native protein (see below) [117]. Finally, the Gly41Ser mutation renders C*c* more susceptible to Met80 oxidation [142].

### 3.7. Homocysteinylation

According to the literature, homocysteinylation of C*c* results from high homocysteine levels in the cell, known as hyperhomocysteinemia [111]. Homocysteine residues bind to the amino group of lysines via amide bonds. In vitro, this PTM is reproduced using homocysteine-tiolactone which reacts spontaneously with free amino groups. The homocysteinylation of lysine amino groups results in a change in the protein surface charge due to the incorporation of homocysteine, which is a less basic amino group. This modification leads to slight changes in the secondary structure which affect the fraction of α-helices [112]. These changes expose some thiol groups, thereby leading to spontaneous formation of protein multimers by intermolecular disulfide bonds [115]. Moreover, the homocysteinylation causes a change in the redox state as a consequence of a homocysteine thiol group incorporation, making the reduced species more thermodynamically stable than the oxidized one and, consequently, homocysteinylated C*c* is more resistant to proteolysis by pronase, trypsin, and chymotrypsin than the non-modified protein [143].

Since lysines are involved in the interaction of C*c* with CIV and C*c*_1_ (from CIII), the homocysteinylation of lysine residues could also affect to the electron transport chain [11,113,134,135,136].

As with sulfoxidation, Sharma et al. found that homocysteinylation by homocysteine tiolactone also confers conformational changes in C*c* which disrupt the heme-Met80 interaction and, consequently, activates C*c* peroxidase activity [114,144], as previously described for the M80A C*c* mutant [137].

### 3.8. Carbonylation

The accumulation of carbonylated proteins has been implicated in cell aging and certain neurodegenerative diseases [145,146]. Carbonylation is an oxidative modification that affects the amino group of lysine residues and is often used as a measure of oxidative damage. Several authors have verified that the carbonylation of C*c* is a consequence of oxidation at other residues, such as Met65/Met80 and Tyr67 or Tyr74, which act as co-activators of lysine carbonylation [12,13,14,147]. Lysine residues are important in the configuration of C*c*. Carbonylation of the Lys72/Lys73 pair impairs its ability to act as heme ligand, thereby generating a free distal site—as it takes place when the Met ligand is already oxidized—that facilitates the entry of peroxide molecules, increasing the peroxidase activity of the hemeprotein [116]. Moreover, the decrease in the positive net charge of Cc impairs its ability to bind CL. Both hallmarks—enhanced peroxidase activity and impaired CL-containing adduct assembly—contribute to the release of C*c* from mitochondria [118]. Unfortunately, specific consequences of C*c* carbonylation on the electron transfer reaction have not been reported in the literature yet.

## 4. Conservation and Evolution of Cytochrome *c* Residues

**Figure 2:**
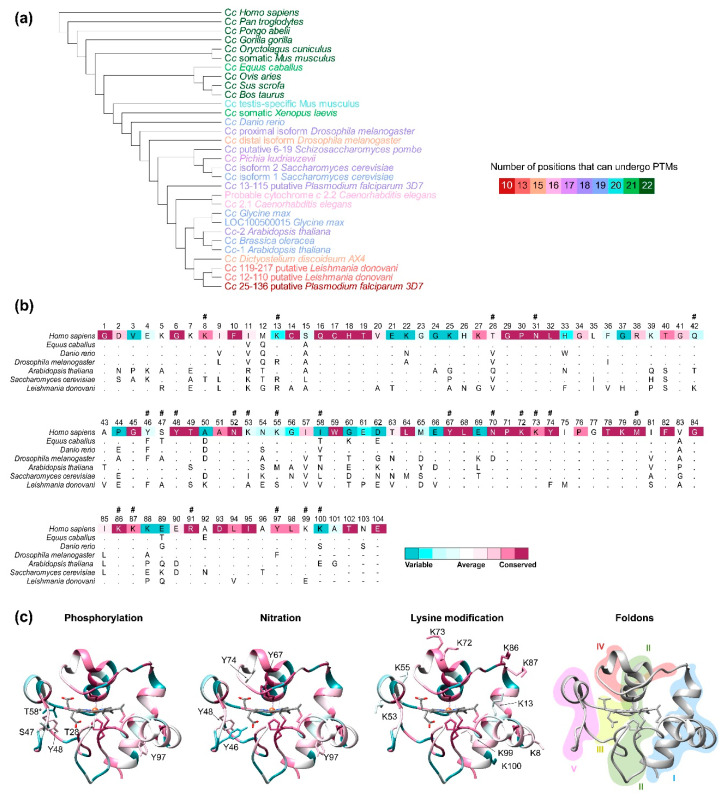
Cytochrome *c* sequence evolutionary conservation and post-translational modifications. (**a**) Evolutionary analysis by Maximum Likelihood method. The evolutionary history was inferred by using the Maximum Likelihood method and General Reversible Mitochondrial + Freq. model [158]. The bootstrap consensus tree inferred from 500 replicates [159] represents the evolutionary history of the taxa analyzed [160]. Branches corresponding to partitions reproduced in less than 50% bootstrap replicates are collapsed. The percentage of replicate trees in which the associated taxa clustered together in the bootstrap test (500 replicates) are shown next to the branches [160]. Initial tree(s) for the heuristic search were obtained automatically by applying Neighbor-Join and BioNJ algorithms to a matrix of pairwise distances estimated using the JTT model, and then selecting the topology with superior log likelihood value. This analysis involved 31 amino acid sequences. There was a total of 122 positions in the final dataset. Evolutionary analyses were conducted in MEGA X [161]. Labels are colored as a function of the number of amino acid positions that can undergo post-translational modifications (PTMs). (**b**) Sequence alignment and amino acid evolutionary conservation of C*c* sequences. Residues are colored according to ConSurf conservation score [162]. Amino acids that can be modified are indicated with (**#**). (**c**) Ribbon representation of human C*c* structure (PDB ID: 2N9I [163]). *Left, r*esidues are colored according to conservation score. Side chain of amino acids that undergo PTMs are labelled. *Right*, the different foldon units are colored as follows: foldon I in blue, foldon II in green, foldon III in yellow, foldon IV in red, and foldon V in purple.

C*c* is a highly conserved protein throughout evolution in terms of functionality. Zaidi and co-workers analyzed the conservation of C*c*-related proteins over 285 unique sequences [148]. Their analysis showed that majority of C*c*-related proteins have a length of 104 amino acids, but only 14% of their residues are conserved along the phylogenetic tree. Given that PTMs finely regulate the functions of C*c*, we aim to analyze whether these conserved residues are targeted for modifications. To achieve this, 31 different respiratory C*c* sequences spanning organisms from five life kingdoms, including model organisms, were analyzed (Figure 2a). Model organisms’ sequences were selected from the landmark BLAST database and sequences of the relevant organisms were manually selected from UniProt database. The analysis revealed that 34% of the residues are highly conserved among the analyzed sequences (Figure 2b). Notably, the number of positions susceptible of PTMs was greater in mammals and closely related species (Figure 2a). C*c* shows a folding model governed by five regions—named foldons—that differ in their stability (Figure 2c) [149,150]. The most stable region is foldon I following by foldon II, the neck or foldon III, the main Ω-loop (foldon IV) and the less stable region, the nested Ω-loop (foldon V). If we focus on the residues that undergo most of the human C*c* PTMs—phosphorylation and nitration of tyrosines, and modifications of lysine residues (Table 1)—we find a correlation with their position and foldon stability (Figure 2c). As previously mentioned, lysine residues play a fundamental role in the interactions of C*c* with its physiological targets, constituting positive patches. Actually, conserved lysine residues undergoing PTMs cluster at the most stable foldon of the protein. The resulting positive surface patch participates in a variety of protein-protein interactions [2,8,9,23,24]. On the other hand, phosphorylated and nitrated residues are mainly in foldons IV and V. The dynamic/flexibility that shows these foldons are substantially affected by PTMs [77,81], modulating the access to heme crevice, which is essential for redox function of C*c*. Other highly conserved residues take part of the heme environment (Cys14, Cys17, Lys72, Lys73, Lys79, and Met80). However, only residues providing the sixth ligand—Met 80 during physiological condition and Lys72, Lys73, or Lys 79 under alkaline transition phenomenon—undergo modifications. This highlights the relevance of the cofactor environment as regards the function of proteins implicated in the redox metabolism [151,152,153,154,155,156,157]. In summary, modulation of C*c* functionality by PTMs is a key factor influencing the evolution of the protein.

## 5. Clinical Relevance of Post-Translationally Modified Cytochrome *c*

**Figure 3:**
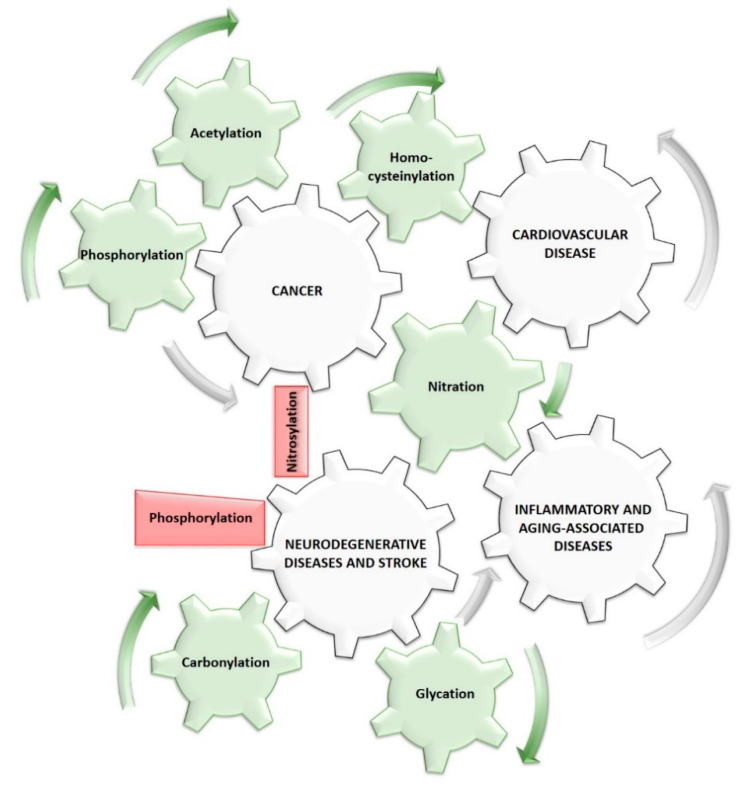
Cause–effect of post-translational modifications of cytochrome *c* in human pathologies. Carbonylation, glycation and nitration of specific residues of C*c* promote the development of several neurodegenerative diseases and strokes. However, phosphorylated C*c* at positions 47 and 97, or nitrosylated in its tyrosine residues has been revealed to be a neuroprotective agent against this type of pathology. Cancer cells show many modifications of heme protein, such as phosphorylations, acetylations, homocysteinilations, and nitrations. Nitrosylation and phosphorylation are mutually exclusive modifications, so the presence of nitrosylated C*c* species could be act as an anticancer agent. Notably, homocysteinilation is also related to cardiovascular diseases. Finally, it has been described that peroxynitrite ion and nitrated proteins (including C*c*) trigger inflammatory processes. The green wheels represent C*c* modifications that favor the development of diseases (see the direction of rotation of the gear indicated by the green arrows), white wheels represent the pathologies—gray arrows represent the direction of rotation that produces the development of the disease—and the red wedges indicate PTMs that impede them.

Proper working of C*c* is essential for cell homeostasis and detoxification. Consequently, its malfunction is related to several mitochondrial diseases and aging [159,164,165]. The cause–effect relationships between post-translationally modified C*c* and several pathologies have been widely studied. Figure 3 graphically summarizes all the described modifications of C*c* that are present in human pathologies. Carbonylation, along with glycation, promote protein aggregation―this includes C*c*, which is one of the principal features in neurodegenerative diseases [99,146]. On the contrary, C*c* phosphorylated in positions Ser47 and Tyr97, or nitrosylated has been revealed as a neuroprotective agent against these neuronal disorders [49,55,81,123]. A plausible explanation is that these modifications inhibit PCD and enhance electron transfer under oxygen deprivation [81]. These findings, along with the fact that non-canonical amino acid-based phosphorylated C*c* mutants are resistant to phosphatases, lead us to propose them as neuroprotectors with promising therapeutic applications. C*c* homocysteinilation occurs in cardiovascular diseases [113] and, together with phosphorylation, in tumors [166]. Cancer cells are characterized by a high growth rate, which derives from enhanced mitochondrial metabolism, as well as an evasion of PCD (Table 1) [131,167]. Bazylianska and co-workers identified the acetylation of Lys53 in C*c* in prostate cancer. In fact, they showed that acetylation strongly inhibits the role of C*c* in apoptosis, allowing the survival of cancer cells [95]. Interestingly, tyrosine nitration and phosphorylation are mutually exclusive modification events. Thus, the Tyr-nitrated C*c* species could function as antagonists to the Tyr-phosphorylated species, acting as an anticancer agent [123]. Moreover, proteins nitrated by peroxynitrite ion (including C*c*) trigger inflammatory processes [168]. Even though C*c* sulfoxidation has been identified in patients with thrombocytopenia, there is no evidence that this PTM directly causes the disease [169].

## Figures and Tables

**Table 1:** Post-translational modifications of cytochrome *c* reported in the literature.

Modification	Sites	Effects	References
Phosphorylation	Thr28, Tyr46, Ser47, Tyr48, Thr58 *, Tyr74, Tyr97	Increase of C*c* peroxidase activity. Decrease of electron transfer efficiency (Thr28, Ser47 and Tyr48). Increase of electron transfer efficiency under supercomplex formation (Tyr97). Modification of redox potential (Tyr48). Inhibition of caspase activation.	[53,55,77,78,79,80,81,82]
Nitration	Tyr46, Tyr48, Tyr67, Tyr74, Tyr97 (only nitration of Tyr74 and Tyr67 are detected in vivo)	Proteolytic degradation (Y46 and Y48). Increase of peroxidase activity (Y46, Y48 and Y74). Inhibition of caspase activation.	[83,84,85,86,87]
Nitrosylation	Heme and Met80	Inhibition of C*c*/CL complex peroxidase activity. Changes in protein conformation and heme coordination	[88,89]
Acetylation	Lysines (only acetylation of Lys8 and Lys53 are detected in vivo)	Decrease of electron transfer efficiency in the respiratory chain. Changes in the protein configuration. Inhibition of caspase activation	[90,91,92,93,94,95,96]
Glycosylation	Lysines ^1^	Down-regulation of proteolytic degradation. Enhancement of thermodynamic stability. Inhibition of caspase activation.	[97,98]
Glycation	Arg91, Lys72, Lys87, Arg92	Monomer aggregation. Reduction of conformational stability. Decrease of electron transfer efficiency in the respiratory chain. Decrease of ability to bind membrane. Enhance peroxidase activity.	[99,100,101,102]
Deamidation	Gln42, Asn31, Asn52 and Asn70	Conformational changes. Modification of redox potential.	[103,104]
Sulfoxidation	Met80	Loss of autoxidizable function. Decrease of electron transfer efficiency in the respiratory chain. Enhance peroxidase activity. Increase of apoptosis induction.	[105,106,107,108,109,110]
Homocysteinylation	Lys8 or Lys13, Lys86 or Lys87, Lys99, and Lys100	Protein denaturation. Increase of resistance to proteolysis. Protein aggregation. Enhancement of peroxidase activity.	[111,112,113,114,115]
Carbonylation	Lys53, Lys55, Lys60 ^†^, Lys72/Lys73	Enhancement of peroxidase activity. Impairment of CL binding. Protein aggregation.	[116,117,118]

* Thr58 is replaced by isoleucine in human C*c*, so its effects are not objective of this study. ^†^ Lys60 is replaced by glycine in human C*c*, so its effects are not objective of this study. ^1^ Unidentified specific modification residues.

## Supplementary Materials

Supplementary materials can be found at https://www.mdpi.com/1422-0067/21/22/8483/s1.

## Abbreviations

AcK Apaf-1	N-ε-acetyl-L-Lys Apoptosis protease-activating factor-1
CL	Cardiolipin
CIII	Complex III
CIV	Complex IV
Cytochrome *c*	C*c*
Cytochrome *c*_1_	C*c*_1_
C*c*O	Cytochrome *c* oxidase
ECT	Electron transport chain
IP3	Inositol 1,4,5-trisphosphate
MAMs	Mitochondria-associated endoplasmic reticulum membranes
PCD	Programmed cell death
*p*CMF pI	*p*-carboxymethyl-L-phenylalanine Isoelectric point
PTMs	Post-translational modifications
RER	Rugose endoplasmic reticulum
RNS	Reactive nitrogen species
ROS	Reactive oxygen species
VDAC	Voltage-dependent anion channel
