# Supplementary material for: Post-Translational Modifications of Cytochrome c in Cell Life and Disease
Source: Int J Mol Sci. 2020 Nov 11;21(22):8483. doi: 10.3390/ijms21228483 (PMC7697256; doi:10.3390/ijms21228483)
Supplement: Supplementary file 1 [file ijms-21-08483-s001.pdf]

International Journal of *Molecular Sciences*

Review

Post-Translational Modifications of Cytochrome *c* in Cell Life and Disease

Alejandra Guerra-Castellano [1], Inmaculada Márquez [1], Gonzalo Pérez-Mejías [1], Antonio Díaz-Quintana [1], Miguel A. De la Rosa [1] and Irene Díaz-Moreno [1,*]

[1] Instituto de Investigaciones Químicas (IIQ), Centro de Investigaciones Científicas Isla de la Cartuja (cicCartuja), Universidad de Sevilla, Consejo Superior de Investigaciones Científicas (CSIC), Avda., Américo Vespucio 49, Sevilla 41092, Spain

* Correspondence: idiazmoreno@us.es

Received: date; Accepted: date; Published: date

Supplementary Materials

Table S1. Non-canonical amino acid

Modified residues	Unnatural amino acid mimic	reference
Phosphotyrosine	4'-carboxymethyloxy-3'-phosphonophenylalanine	[1]
	N-[4-(2-{2-[3-(2-Bromo-acetylamino)-propionylamino]-3-hydroxy-propionylamino}-ethyl)-phenyl]-oxalamic acid	[2]
	3-(4-{2-[2-(2-bromo-acetylamino)-ethyldisulfanyl]-ethylcarbamoyl}-cyclohexylcarbamoyl)-pyrazine-2-carboxylic acid	[2]
	Photocaged phosphotyrosine	[3]
	Sulfotyrosine	[4]
	p-carboxymethyl-L-phenylalanine	[5]
	p-(phosphonoamino)-phenylalanine	[6]
	O-phosphotyrosine	[7]
	Benzyl 2-benzyloxycarbonylamino-3-[4-bis(dimethylamino)phosphoryloxyphenyl] propanoate	[8]
	4-phosphomethyl-L-phenylalanine	[9]
	p-azido-L-phenylalanine	[10]
Phosphothreonine	Photocaged phosphothreonine	[3]
	O-phospho-L-threonine	[11]
Phosphoserine	Photocaged phosphoserine	[3]
	Phosphonomethylene alanine	[12]
Nitrotyrosine	3-nitrotyrosine	[13]
Acetyl-lysine	N^{ε}-acetyl-lysine	[14]
	Trifluoracetil-lysine	[15]
	2-amino-8-oxononanoic	[16]

References

1. Kawahata, N.; Yang, M.G.; Luke, G.P. A novel phosphotyrosine mimetic 4′-carboxymethyloxy-3′-phosphonophenylalanine (Cpp): exploitation in the design of nonpeptide inhibitors of pp60(Src) SH2 domain. *Bioorg Med Chem Lett* **2001**, *11*, 2319–2323.
2. Erlanson, D.A.; McDowell, R.S.; He, M.M. Discovery of a new phosphotyrosine mimetic for PTP1B using breakaway tethering. *J Am Chem Soc* **2003**, *125*, 5602–5603.
3. Rothman, D.M.; Petersson, E.J.; Vázquez, M.E. Caged phosphoproteins. *J Am Chem Soc* **2005**, *127*, 846–847.
4. Liu, C.C.; Schultz, P.G. Recombinant expression of selectively sulfated proteins in *Escherichia coli*. *Nat Biotechnol* **2006**, *24*, 1436–1440.
5. Xie, J.; Supekova, L.; Schultz, P.G. A genetically encoded metabolically stable analogue of phosphotyrosine in *Escherichia coli*. *ACS Chem Biol* **2007**, *2*, 474–478.
6. Serwa, R.; Wilkening, I.; Del Signore, G. Chemoselective Staudinger-phosphite reaction of azides for the phosphorylation of proteins. *Angew Chem Int Ed Engl* **2009**, *48*, 8234–8239.
7. Fan, C.; Ip, K.; Söll, D. Expanding the genetic code of *Escherichia coli* with phosphotyrosine. *FEBS Lett*. **2016**, *590*m 3040–3047.
8. Hoppmann, C.; Wong, A.; Yang, B. Site-specific incorporation of phosphotyrosine using an expanded genetic code. *Nat Chem Biol* **2017**, *13*, 842–844.
9. Luo, X.; Fu, G.; Wang, R.E. Genetically encoding phosphotyrosine and its nonhydrolyzable analog in bacteria. *Nat Chem Biol* **2017**, *13*, 845–849.
10. Chin, J. W.; Santoro, S. W.; Martin, A. B.; King, D. S.; Wang, L.; Schultz, P. G. Addition of *p*-azido-L-phenylaianine to the genetic code of *Escherichia coli. J Am Chem Soc* **2002**, **124**, 9026–9027.
11. Zhang, M.S.; Brunner, S.F.; Huguenin-Dezot, N. Biosynthesis and genetic encoding of phosphothreonine through parallel selection and deep sequencing. *Nat Methods* **2017**, *14*, 729–736.
12. Rogerson, D.T.; Sachdeva A.; Wang, K. Efficient genetic encoding of phosphoserine and its nonhydrolyzable analog. *Nat Chem Biol* **2015**, *11*, 496–503.
13. Sokolovsky, M; Riordan, J.F., Vallee, B.L. Conversion of 3-nitrotyrosine to 3-aminotyrosine in peptides and proteins. *Biochem Biophys Res Commun* **1967**, *27*, 20-25.
14. Neumann H, Peak-Chew SY, Chin JW. Genetically encoding N(epsilon)-acetyl-lysine in recombinant proteins. *Nat Chem Biol* **2008**, *4*, :232-234.
15. Hancock, S. M.; Uprety, R.; Deiters, A.; Chin, J. W. Expanding the genetic code of yeast for incorporation of diverse unnatural amino acids via a pyrrolysyl-tRNA synthetase/tRNA pair. *J Am Chem Soc* **2010**, *132*, 14819–14824.
16. Huang, Y; Wan, W; Russell, W.K.; Pai, P. J.; Wang, Z.; Russell, D. H.; Liu, W. Genetic incorporation of an aliphatic keto-containing amino acid into proteins for their site-specific modifications. *Bioorg Med Chem Lett* **2010**, *20*, 878-880.

© 2020 by the authors. Submitted for possible open access publication under the terms and conditions of the Creative Commons Attribution (CC BY) license (http://creativecommons.org/licenses/by/4.0/).
